# Allele Mining and Selective Patterns of *Pi9* Gene in a Set of Rice Landraces from India

**DOI:** 10.3389/fpls.2016.01846

**Published:** 2016-12-15

**Authors:** Jahangir Imam, Nimai P. Mandal, Mukund Variar, Pratyoosh Shukla

**Affiliations:** ^1^Biotechnology Laboratory, Central Rainfed Upland Rice Research StationHazaribagh, India; ^2^Enzyme Technology and Protein Bioinformatics Laboratory, Department of Microbiology, Maharshi Dayanand UniversityRohtak, India

**Keywords:** allele mining, rice landraces, polymorphism, blast resistance genes, selection pressure

## Abstract

Allelic variants of the broad-spectrum blast resistance gene, *Pi9* (nucleotide binding site-leucine-rich repeat region) have been analyzed in Indian rice landraces. They were selected from the list of 338 rice landraces phenotyped in the rice blast nursery at central Rainfed Upland Rice Research Station, Hazaribag. Six of them were further selected on the basis of their resistance and susceptible pattern for virulence analysis and selective pattern study of *Pi9* gene. The sequence analysis and phylogenetic study illustrated that such sequences are vastly homologous and clustered into two groups. All the blast resistance *Pi9* alleles were grouped into one cluster, whereas *Pi9* alleles of susceptible landraces formed another cluster even though these landraces have a low level of DNA polymorphisms. A total number of 136 polymorphic sites comprising of transitions, transversions, and insertion and deletions (InDels) were identified in the 2.9 kb sequence of *Pi9* alleles. Lower variation in the form of mutations (77) (Transition + Transversion), and InDels (59) were observed in the *Pi9* alleles isolated from rice landraces studied. The results showed that the *Pi9* alleles of the selected rice landraces were less variable, suggesting that the rice landraces would have been exposed to less number of pathotypes across the country. The positive Tajima’s D (0.33580), *P* > 0.10 (not significant) was observed among the seven rice landraces, which suggests the balancing selection of *Pi9* alleles. The value of synonymous substitution (-0.43337) was less than the non-synonymous substitution (0.78808). The greater non-synonymous substitution than the synonymous means that the coding region, mainly the leucine-rich repeat domain was under diversified selection. In this study, the *Pi9* gene has been subjected to balancing selection with low nucleotide diversity which is different from the earlier reports, this may be because of the closeness of the rice landraces, cultivated in the same region, and under low pathotype pressure.

## Introduction

Rice blast (*Magnaporthe oryzae*), the most serious diseases of rice causes significant yield loss globally and the complexity of pathogen, host, and microclimate have a profound effect on this (Valent, 1990; Teng et al., 1991; Kwon and Lee, 2002; Li et al., 2007). The blast fungus is both sexual and asexual in nature which resulted in the evolution of its different variants in field conditions (Xia et al., 1993). The high adaptation frequency and variations lead to the emergence of new races of the fungal population of *M. oryzae* leads to the breakdown of resistance in newly released rice cultivars in the fields (Kiyosawa et al., 1986; MacKill and Bonman, 1992; Valent and Chumley, 1994; Han et al., 2001). Under normal field condition, incomplete, or field resistance of blast disease is better options for the effective control of *M. oryzae* (Liu et al., 2005). The host–pathogen interaction can be better understood by the identification and characterization of both *R* and *Avr* genes and certain enzyme based studies on technological improvements through combined approaches (Imam et al., 2014a,d, 2015a, 2016; Baweja et al., 2016; Kumar et al., 2016). Till date, many blast resistance genes and QTLs have been recognized and cloned (Sharma et al., 2005; Imam et al., 2014b, 2015b). Most of the rice blast resistance genes cloned till date encode nucleotide binding site-leucine-rich repeat (NBS-LRR) proteins which suggest the common escape root involving a familiar resistance pathway to counter blast infections (Hammond-Kosack and Jones, 1997; Takken and Tameling, 2009; Liu et al., 2010; Imam et al., 2013b).

The transfer of valuable alleles found in the rice germplasm is generally employed by the plant breeders for the improvement of high-yielding varieties (Kumar et al., 2010). Natural mutation like transition, transvertion, point mutation, and insertion and deletions (InDels) is the main driving force for the generation and evolution of new alleles. With the availability of enormous database information, desired and superior alleles can be easily identified and retrieved (Kumar et al., 2010). The potential application of allele mining approach is in the identification of new haplotypes and evolution pattern study which helps in the rice improvement programs (Kumar et al., 2010). TILLING (Targeting Induced Local Lesions in Genomes) and sequence-based allele mining are the two main approach for sequence polymorphism study in the natural population of germplasm (Till et al., 2003; Kumar et al., 2010). Allele mining has emerged as an important approach for cloning and characterization of new and better forms of disease resistance genes. Isolation of orthologs provides insights into the evolutionary forces shaping the development that help identification of better alleles for future experiments (Ashkani et al., 2015). Because of its facile nature, this approach is being used extensively to identify alleles of agriculturally important traits. Wild as well as cultivated rice varieties has been studied for the blast resistance genes by allele mining approach (Geng et al., 2008; Huang et al., 2008; Yang et al., 2008). An extensive study of the *Pi-ta* locus was described from wild species of rice (Huang et al., 2008), cultivated (AA) and wild species and invasive weedy rice (Lee et al., 2009, 2011). Another gene, *Pid3* studied from 36 rice lines of both cultivated and wild species indicated pseudogenization of *Pid3* in *japonica* cultivars (Shang et al., 2009). Liu et al. (2011) reported divergent selection in *Pi9* locus cloned from cultivated and wild species of rice. Most of the above mentioned blast genes were studied through sequence-based allele mining approach.

The *Pi9, Pi2*, and *Piz-t*, the three paralogs of the blast resistance gene family is now well characterized and resistance mechanism is known (Qu et al., 2006; Zhou et al., 2006). MacKill and Bonman (1992) discussed the origins of *Piz-t* and *Pi2* genes from *indica* cultivars while *Oryza minuta*, a wild rice is the source of origin of *Pi9* gene and high LRR positive selection (Amante-Bordeos et al., 1992; Zhou et al., 2007; Dai et al., 2010). The *Pi9* locus contains at least six known resistance genes specific to the fungal pathogen *M. oryzae* and three *R*-genes from this locus (*Pi9, Pi2*, and *Piz-t*) have been cloned (Qu et al., 2006; Zhou et al., 2006). The resistance specificities of different broad spectrum rice blast resistance genes shown to be different from one another which mainly arise because of the different evolutionary changes in the NBS-LRR genes and its generation and also the helps in the adaptation to ever changing pathogen populations (Bergelson et al., 2001; Shen et al., 2006; Liu et al., 2007, 2013; Yang et al., 2008). For the preservation of the resistant germplasm, knowledge of the variation patterns of *R*-genes is important. Yang et al. (2006) worked on the genome-wide allelic analysis of *R*-genes between two rice cultivars and categorize the variation patterns into four types, namely type I, type II, type II, and type IV from conserved to presence/absence genes. Basically type I and type II plays the main role in *R*-genes allelic polymorphism and resistance specificity and this give rise to rapid evolution in these blast resistance genes and enable them to adapt to the ever-changing pathogen population (Bergelson et al., 2001; Shen et al., 2006; Yang et al., 2008). The single-copy gene dominant group (type I), showed the lowest diversity (<0.005); the clustered-gene dominant group (type III), have a high level of diversity and the intermediate one (type II), and the presence or absence of *R*-genes in one genomes (P/A *R-*genes, type IV) (Yang et al., 2008). Different blast resistance genes showed different levels of polymorphism and diversity. The LRR region of *Pi54* and *Piz-t* genes are more vulnerable for changes and leads to positive directional selection as compared to LRR regions encoded by *Pi-km1* and *Pi-km2* blast ressitance genes which are highly conserved (Ashikawa et al., 2010; Thakur et al., 2013). It is interesting to note that LRRs have direct interacting roles with effector proteins (Young and Innes, 2006). Higher levels of polymorphism were observed in the LRR region of *Pi54* which helps in effector recognition and the evolutionary pressure by virulent *M. oryzae* races results in in variations in the LRR domain (Thakur et al., 2015).

Allelic polymorphisms in the *R-*genes are mainly driven by balancing selection and positive selection. The positive selection is the main evolutionary force which maintains the polymorphisms in the *R*-genes family plants (Bent and Mackey, 2007; Zhou et al., 2007). The genome structure at *Pi9* locus is highly conserved but the LRR region showed high sequence variation giving rise to positive selection for *Pi9* genes among the rice germplasm (Zhou et al., 2007; Dai et al., 2010; Liu et al., 2010). Previous data toward the prevalence of high pathotypic diversity of the *M. oryzae* population from Eastern India and very rare compatibility of *Pi9* gene in field evaluations with isolates from this region provoked this study (Variar et al., 2009). Amongst the multi-genes near the waxy gene locus on chromosome 6, *Pi9* was extra efficient than *Pi2* (*Piz-t*) in the preceding investigation (Ballini et al., 2008; Imam et al., 2014b). Our recent studies on the rice and *M. oryzae* interaction of isolates collected from India reveals the compatible and incompatible interactions between the *R* and the corresponding *Avr* genes (Imam et al., 2013b, 2014a, 2015a). Despite the fact that resistance mediated by single *R-*gene can be easily wrecked by emerging virulence, some cultivars with major resistance genes have stay resistant for a extended time without resistance loss (Khush and Jena, 2009). A likely rationale for the durability of *Pi9-*mediated resistance to blast is the fact that the gene presents broad-spectrum resistance to miscellaneous isolates. The germplams harboring the *Pi9* gene identified in the earlier study originated from different Eastern Indian locations exhibited excellent resistance to several isolates from the region, which is appealing to hypothesize that the gene is effective and durable (Imam et al., 2014c). To better understand the genetic polymorphism and molecular evolution mechanism of the *Pi9* alleles, we analyzed the 2.9 kb region of the *Pi9* gene in six accessions of cultivated rice landraces. Therefore, the present investigation is taken up for the allele mining of NBS-LRR region of *Pi9* gene from the rice landraces to better understand the sequence polymorphisms and its relevance in resistance and susceptibility pattern. The objectives of this study were (1) to isolate alleles of *Pi9* blast resistance gene, (2) to understand the nucleotide diversity in NBS-LRR region of *Pi9* gene, and (3) analysis of the molecular evolution and patterns of selection in this region.

## Materials and Methods

### Plant Materials

A selected set of 338 rice landraces accessions which were re-evaluated in the uniform blast nursery (UBN) were considered for allele mining of *Pi9* gene. Out of 338, seven rice landraces accessions were taken for further analysis and allele mining (**Table 1**). The selection for allele mining was based on the result of the presence of *Pi9* gene by STS marker and their disease score. A pair of dominant STS markers 195R-1 (5′-ATGGTCCTTTATCTTTATTG-3′) and 195F-1 (5′-TTGCTCCATCTCCTCTGTT-3′) derived from the *Nbs2-Pi9* candidate gene was used to check the presence of *Pi9* gene in the rice landraces in this study (Qu et al., 2006). Out of seven, one is the iso-genic line for *Pi9* gene, three were resistant and rests three were susceptible to blast disease.

**Table 1:** Rice landraces sourced from NBPGR and their reaction to Magnaporthe *oryzae* at uniform blast nursery (UBN), Hazaribag selected for allele mining of *Pi9* gene.

S.No.	Name of rice landraces	Id	Type	Classification	Source	State/Origin	Phenotype (R/S)
1	IRBL9-w	LRI_1	Japonica	Iso-genic line (Control)	IRRI	IRRI	R
2	IC450108	LRI_2	Indica	Landrace	DRR	India	R
3	IC449579	LRI_3	Indica	Landrace	DRR	India	R
4	IC347244	LRI_4	Indica	Landrace	DRR	India	R
5	IC449609	LRI_5	Indica	Landrace	DRR	India	S
6	IC449695	LRI_6	Indica	Landrace	DRR	India	S
7	IC450033	LRI_7	Indica	Landrace	DRR	India	S

### Phenotype Evaluation of Landraces

A mixture of virulent isolates (Mo-ei-66, Mo-ei-79, Mo-ei-119, and Mo-ei-202) was used as inoculum for the phenotyping of selected rice landraces (Imam et al., 2013a). Oat Meal Agar (HiMedia, India) medium was used to maintain the fungal culture of each isolate. The Mathur’s medium was used for the sporulation and multiplication of fungal spores. These cultures were preserved at 22°C for 12–16 days under stable illumination from white fluorescent light (55 μF/Em/s) (Thakur et al., 2013). Conidia were split from the conidiophores which were used for the preparation of fungal spores and the inoculum were maintained to approximately 10^5^ spores/ml. The leaf stage seedlings (2–3 in number) in replicated sets were spray-inoculated with 1 ml mixed spore suspension and then kept back in darkness at 27°C and over 90% relative humidity for 24 h. In this experiment, positive control for *Pi9* gene (IRBL9-w) and rice landraces was grown in plastic pots and maintained in phenotyping facility. After inoculation with mixed fungal cultures, the rice seedlings were maintained in the phenotyping chamber with desired temperature and humidity. Analysis of virulence was completed on the basis of reaction type using 0–5 standard evaluation scale. Resistance was scored based on no visible infection and no conidia produced from inoculated tissue (scores 0, 1, 2), while susceptibility was scored with a lesion >3 mm in length (score 3, 4, 5) and sporulating (Bonman et al., 1986).

### PCR and Sequencing

Overlapping oligos were designed using Primer 3 software ^1^ to amplify 2.9 kb NBS-LRR region of *Pi9* gene (DQ285630) using primer walking technique (Thakur et al., 2015). A total of five primer pairs was designed to amplify the 2.9 kb region (**Table 2**). PCR was carried out from the isolated DNA of the iso-genic line *IRBL9-w* and six rice landraces using Q5 high-fidelity DNA polymerase (New England Biolabs, Life Technologies, USA) to amplify full-length allele with high-fidelity with the following thermal cycling conditions: initial DNA denaturation at 95°C for 2 min followed by 30 cycles of 95°C for 30 s, 58°C for 30 s, 72°C for 1 min, final elongation at 72°C for 10 min and hold at 48°C. The amplified PCR products were then sequenced ^2^ and assembled. Phred/Phrap/Consed software (Ewing and Green, 1998) was used for the assembly of multiple reads of different fragments to form the full-length allele. For data analysis good quality (>Phred Phred 30) consensus sequence was used.

**Table 2:** List of overlapping primers used for the amplificati1on of 2.9 kb nucleotide binding site-leucine-rich repeat (NBS-LRR) region of *Pi9* gene using primer walking technique.

S.No.	Fragments	Forward Primer (5′–3′)	Reverse Primer (5′–3′)	Tm (°C)	Size (bp)
1	*Pi9*_1	ATCAGTCACAAAATAGACTGTCATG	AGAGCTGTCTTGCCTAAACCAC	60.0	741
2	*Pi9*_2	GATGGGTGGTTTAGGCAAGACAG	CGACATCCTTAGTCGTCATC	60.0	836
3	*Pi9*_3	TCGTCTAGTAGGTAGATGGATAGCAG	TACCTTCACACCGAATGATTCAG	60.0	808
4	*Pi9*_4	ACCAAAGTTGCTGGTCTGAATCA	TCGTGATCCCTTCGGTCACTGTC	60.0	791
5	*Pi9*_5	GTGCTGCGAATGGACAGTGACCGA	CTGATCTCATATTCCCTTCCACAAT	60.0	742

### Sequence Analysis

Alignment of assembled sequences and manual editing of blast resistance gene *Pi9* was done by ClustalW (Thompson et al., 1994) and BioEdit Software version 7.0.9.0 ^3^. *Pi9* gene sequence (DQ285630) was used as a reference for the prediction of gene coding regions by using Gene FGENESH ^4^. The functional domain(s) which play an important role in mediating resistance were predicted using the online tools Pfam) ^5^ and SMART ^6^. Phylogenetic analysis was performed with MEGA 4.0 (Tamura et al., 2007) using the Neighbor-Joining method (Saitou and Nei, 1987). All positions containing gaps and missing data were eliminated from the dataset (complete deletion option).

### Nucleotide Polymorphisms Analysis

Nucleotide polymorphism analysis of the aligned DNA sequences was done by DnaSP 5.10 program (Rozas et al., 2003). The Dna SP 5.10 program was used for the analysis of polymorphisms and Tajima’s *D* test. The BioEdit software was used to calculate pairwise identity at DNA level. Synonymous and non-synonymous substitution (π_syn_ and π_non_) were calculated to examine the selection at the NBS-LRR region of *Pi9* gene.

## Results

### Selection of Rice Landraces and Virulence Analysis

**FIGURE 1:**
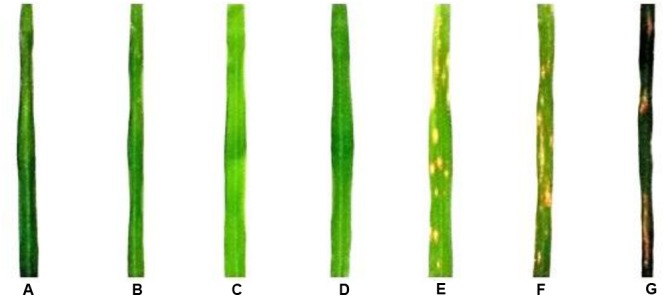
**Pathogenicity assay of *Pi9* positive rice landraces. (A)**, IRBL9-w; **(B)**, IC450108; **(C)**, IC449579; **(D)**, IC347244; **(E)**, IC449609; **(F)**, IC449695; **(G)**, IC450033.

On the basis of pathotyping of 338 rice landraces at UBN, Hazaribag, six landraces comprising of three resistant and susceptible each, was selected for the allele mining of NBS-LRR region of *Pi9* gene (**Table 1**). To further confirm their resistance and susceptibility, these rice landraces were along with the isogenic line for *Pi9* gene IRBL9-w (control) were phenotyped with the mixture of virulent isolates discussed earlier (Imam et al., 2013a). The virulence analysis results showed that, out of six landraces, three were consistently resistant while three showed susceptibility to the mixture of virulent *M*. *oryzae* isolates (**Figure 1**). IRBL9-w, the isogenic lines for *Pi9* gene was also given a resistant reaction.

### Sequence Characterization of the *Pi9* Alleles

**FIGURE 2:**
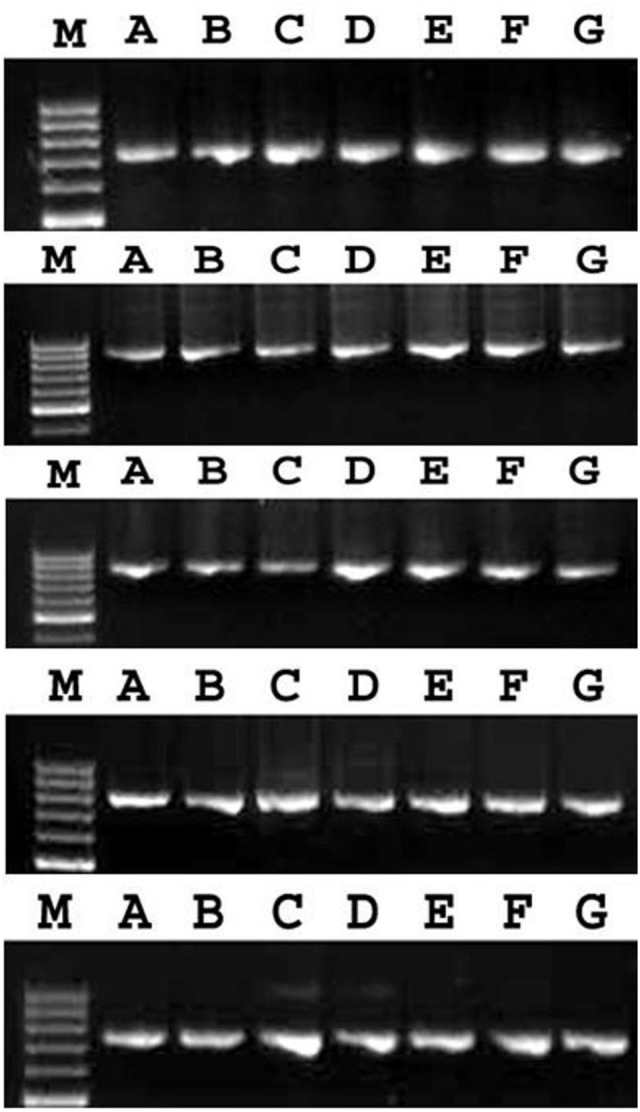
**PCR amplification of different fragments of NBS-LRR region of *Pi9* gene from positive control, IRBL9-w (A) and six rice landraces (B–G).** M, Molecular marker (1 kb) and lane A to G, PCR fragment obtained from overlapping fragments of *Pi9* allele. *Pi9*_1 primer (740 bp), *Pi9*_2 primer (836 bp), *Pi9*_3 primer (808 bp), *Pi9*_4 primer (791 bp), *Pi9*_5 primer (742 bp).

**FIGURE 3:**
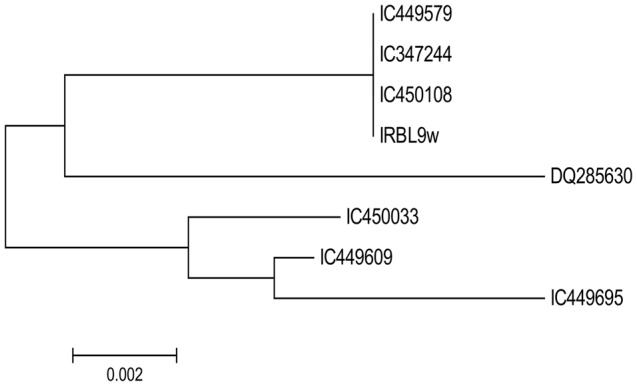
**Phylogenetic tree of *Pi9* allele based on nucleotide sequences of seven rice landraces along with one reference sequence**.

To determine the nucleotide diversity at the *Pi9* allele, 2.9 kb long fragment were amplified from all the seven rice landraces by primer walking technique and sequenced (**Figure 1**). Only high-quality reads of the sequenced fragments were selected for analysis. About 99% (98%–100%) homology between the sequences was observed after pairwise alignment at the DNA level. Lower variation in the form of mutations (77) (Transition + Transversion), and InDels (59) was observed in the *Pi9* alleles isolated from rice landraces selected. The phylogenetic tree was constructed based on the nucleotide sequences of seven rice landraces and one reference *Pi9* (DQ285630) sequence (**Figures 2** and **3**). Phylogenetic analysis results in the formation of two groups, which clearly demonstrate the homology between the sequences. All the blast resistance *Pi9* alleles were grouped into one cluster, whereas *Pi9* alleles of susceptible landraces formed another cluster even though these landraces have a low level of DNA polymorphisms.

### Nucleotide Polymorphism of the *Pi9* Alleles

A total number of 75 polymorphic sites were identified in the 2.9 kb sequence among all the *Pi9* alleles using DnaSP program. Average pairwise nucleotide diversity (π) and silent Watterson’s nucleotide diversity estimator (𝜃_w_) over the *Pi9* alleles were 0.01103 and 0.01011, respectively. The average number of nucleotide differences, *k* was 31.536 and 𝜃 (per site) from Eta was 0.01038. Low-diversified nucleotide diversity for *Pi9* alleles was observed based on earlier published results. The results showed that the *Pi9* alleles of the selected rice landraces were less variable, suggesting that these rice landraces would have been exposed to less number of pathotypes across the country. LRR region showed higher average nucleotide diversity than that of the NBS region and this clearly suggests the importance of LRR domain in the variation of the *Pi9* alleles.

### Selection of *Pi9* Alleles

We evaluated the neutral selection with the Tajima’s *D* test to test the evolutionary selection dynamics of *Pi9* alleles in the rice lanraces. Among the seven rice landraces, positive Tajima’s *D* (0.33580) was observed, which signifies the balancing selection among them, which is different from the earlier results (Tajima, 1989). The presence of less number of rare alleles may be the reason for the positive Tajima’s *D* test. Average rates of non-synonymous and synonymous substitution (π_syn_ and π_non_) were calculated to examine the selective patterns of *Pi9* gene in the rice landraces. The synonymous (π_syn_) and non-synonymous (π_non_) substitution in coding region as a whole were calculated in all the seven *Pi9* alleles. In the coding region, the value of synonymous substitution (-0.43337) was less than the non-synonymous substitution (0.78808). The greater non-synonymous substitution than the synonymous means that the coding region, mainly the LRR domain was under diversified selection. The Tajima’s *D* ratio (Non-syn/Syn) was -1.81851 (<1), suggesting the low level of polymorphism in the coding regions of rice landraces. A haplotype distribution analysis was done for all the seven alleles to study mutations and polymorphisms. The study of sequence polymorphism leads to the identification of a total number of five (5) haplotypes (**Table 3**).

**Table 3:** List of identified haplotypes.

Haplotypes	No. of *Pi9* alleles	Name of *Pi9* alleles
Hap_1	1	[DQ285630]
Hap_2	4	[IRBL9-w, IC450108 IC449579 IC347244]
Hap_3	1	[IC449609]
Hap_4	1	[IC449695]
Hap_5	1	[IC450033]

## Discussion

The analysis of allelic variants of disease resistance gene imparts essential information regarding novel resistance gene generation and specificity. Earlier reports showed both higher as well as lower levels of sequence diversity at different *R*-gene locus (Yang et al., 2008). In this study, polymorphism of the *Pi9* allele was investigated in seven rice landraces. Earlier study about the prevalence of high pathotypic diversity of the *M. oryzae* population of Eastern India and very rare compatibility of *Pi9* gene in field evaluations with isolates from this region result in considering *Pi9* gene further in rice landraces (Variar et al., 2009). Our earlier results of virulence analysis of 72 *M. oryzae* isolates against 26 differential variety revealed that matching virulence to all monogenic differentials carrying different resistant genes were present in the pathogen population, although the resistant check Tetep was resistant to all of them. The frequency of virulence on different monogenic lines ranged from 4.5 to 73%. Very low frequencies of isolates were virulent on *Pi9* (4.5%) and *Piz-5(Pi-2*) (7%) followed by *Pita-2* (16 and 18.2%) were reported (Alam et al., 2015). Therefore, complementary resistance spectra that exclude all the pathotypes of the pathogen are required for strategic resistant gene deployment. *Pi9* and *Pita-2* genes exhibited complementary resistance spectrum and excluded all the pathotypes of the pathogen. Therefore, *Pi9* was taken into consideration for further study and analysis. The present result showed that the alleles of the rice landraces were mostly identical at the DNA sequence level, which further suggests the high level of conservation among *Pi9* rice germplasms. A total number of 136 polymorphic sites comprising of transitions, transversions, and InDels were identified in the 2.9 kb sequence of *Pi9* alleles. Simple InDels and Single nucleotide polymorphisms (SNPs) play a very important function in *R*-gene evolution (Shen et al., 2006). A single nucleotide difference in the regulatory region of *Pi54* locus distinguishes resistant phenotype from the susceptible one (Sharma et al., 2005). The *Pita* gene when physically linked to a region called superlocus able to show resistance pattern (Jia and Martin, 2008; Lee et al., 2009). In cereal genomes, higher SNPs are detected in the in non-coding regions (one in 100–600 bp) (Gupta and Rustogi, 2004). Similarly, between *O. sativa* and *O. rufipogon*, the 26 kb region of DNA sequence showed higher variation (Rakshit et al., 2007). The results clearly showed 99% similarity and low polymorphism at the DNA level among all the seven rice landraces, however, presence of SNPs make it little variable at some regions. The low polymorphism in the DNA sequences of rice landraces reveals that these landraces are closely related were exposed to less number of pathotypes. Liu et al. (2011) demonstrated the intermediate level of polymorphism of the *Pi9* alleles from 40 *Oryza* accessions of China are belonging to cultivate and wild species.

On the basis of a genome-wide analysis of allelic diversity in *R*-genes of the rice genome, four classes of diversification of *R*-genes are described (Yang et al., 2008). The present study with seven rice landraces indicated that *Pi9* allele belongs to type II category since it was neither highly conserved not highly diverse, even though it has low diversified alleles, similar to other blast resistance gene *Pi54* (Thakur et al., 2015). Different studies showed that the rapid evolution of *R*-genes are driven by the high level of diversification (Type III and Type IV) and polymorphism (Shen et al., 2006; Yang et al., 2008; Liu et al., 2011). Pairwise allelic diversity, genomic organization, and the genealogical relationship among different genes have been the criteria to characterize the variation patterns which results in the categorization in four types of variation. Our studies also demonstrateed similar diversification of conserved (Type I; π < 0.5%), highly diversified (Type III; π > 0.5%), intermediated-diversified (Type II; π = 0.5-5%) and present/absent genes (Type IV) as previously published reports (Yang et al., 2008; Liu et al., 2011). Earlier study by Liu et al. (2011) suggest that both human and natural selection played a major role in evolutionary divergence of the *Pi9* gene after the rice species differentiation. The allelic variation among the rice germplasm in the NBS-LRR region has increased our understanding of variation patterns. Earlier studies showed that variation level of *R*-gene was generally constant among the rice germplasms. This is now believed that the polymorphism content directly correlates to evolutionary changes (Shen et al., 2006; Yang et al., 2008). For the *R-*gene resistance specificity, LRR region is the major determinant which is largely responsible for the variation in the NBS-LRR genes (Collier and Moffett, 2009). It is also inetersting to note that among and within *oryza* species (wild and cultivated rice), LRR region showed more sequence variation than NBS region (Liu et al., 2011). Since *Pi9* alleles showed Type II intermediate level of polymorphism, therefore, its evolution pattern is slow and intermediate during the course of time (Ding et al., 2007; Yang et al., 2008). The present study suggests the intermediate level of polymorphism in the *Pi9* alleles which may be due to the mixed evolutionary pressure experienced by the gene during co-evolution of rice blast pathogen. In another study, among the cultivated rice, the *Pita* alleles showed the lowest rate of diversification as among other rice species (Lee et al., 2009). Low nucleotide variation was observed in the coding region (0.00067) of *Pita* alleles in US weedy rice as compared to non-coding regions (0.00161) (Lee et al., 2011). Interestingly, the phylogenetic analysis showed that resistant and susceptible *Pi9* alleles grouped into separate clusters. This is in line to *Pi9* alleles wherein cultivated rice along with its ancestors clustered into one group and African cultivated rice along with its ancestors grouped into separate cluster, suggesting that same selection pressure has occurred in two groups during domestication and/or natural selection (Liu et al., 2011). Thakur et al. (2013) also demonstrated the grouping of resistant and susceptible *Piz-t* alleles in two sub-cluster. In *R*-gene evolution and development of resistance specificity, the LRR region plays the major role (Collier and Moffett, 2009). The present result also showed the high level of sequence variation in LRR region among the rice landraces.

## Conclusion

In *R*-gene evolution, balancing as well as positive selection has been observed and different test is used to calculate the selection pressure which drives the evolution of *R*-genes (Hudson et al., 1987; Tajima, 1989; McDonald and Kreitman, 1991). In this study, it appears to be balancing selection because of the minor positive Tajima’s *D* test value, which is different from the earlier reports of Liu et al. (2011), which showed that the *Pi9* gene is under positive selection. The reason for having positive Tajima’s *D* test was the low nucleotide diversity within the rice germplasm. This may be because of the closeness of the rice landraces, cultivated in the same region, and under low pathotype pressure. The Tajima’s *D* ratio (Non-syn/Syn) is an indicative of selection pressure acting on the protein coding genes. Both balancing and purifying selections have been observed for the evolution of *R*-gene (Thakur et al., 2013).

## Author Contributions

All authors listed, have made substantial, direct and intellectual contribution to the work, and approved it for publication.

## Conflict of Interest Statement

The authors declare that the research was conducted in the absence of any commercial or financial relationships that could be construed as a potential conflict of interest.

## Footnotes

1 www.bioinfo.ut.ee

2 www.chromous.com

3 www.mbio.ncsu.edu

4 www.softberry.com

5 pfam.xfam.org

6 smart.embl-heidelberg.de

## References

[B1] Alam S. Imam J. Nitin M. Prasad C. Variar M. (2015). Molecular screening of Blast resistance gene Pi2 in Indian rice landraces (*Oryza sativa* L.) and its verification by virulence analysis. *Proc. Natl. Acad. Sci. India Sect. B Biol. Sci.* 10.1007/s40011-015-0548-3

[B2] Amante-Bordeos A. Sitch L. A. Nelson R. Damacio R. D. Oliva N. P. Aswidinnoor H. (1992). Transfer of bacterial blight and blast resistance from the tetraploid wild rice *Oryza minuta* to cultivated rice, *Oryza sativa*. *Theor. Appl. Genet.* 84 345–354. 10.1007/BF00229493 24203194

[B3] Ashikawa I. Wu J. Matsumoto T. Ishikawa R. (2010). Haplotypediversity and molecular evolution of the rice Pikm locus for blast resistance. *J. Gen. Plant Pathol.* 76 37–42. 10.1007/s10327-009-0213-x

[B4] Ashkani S. Yusop M. R. Shabanimofrad M. Azadi A. Ghasemzadeh A. Azizi P. (2015). Allele mining strategies: principles and utilization for blast resistance gene in rice (*Oryza sativa* L.). *Curr. Issues Mol. Biol.* 17 57–74. 25706446

[B5] Ballini E. Morel J. B. Droc G. Price A. Courtois B. Notteghem J. L. (2008). A genome-wide meta-analysis of rice blast resistance genes and quantitative trait loci provides new insights into partial and complete resistance. *Mol. Plant Microbe Interact.* 21 859–868. 10.1094/MPMI-21-7-0859 18533827

[B6] Baweja M. Nain L. Kawarabayasi Y. Shukla P. (2016). Current technological improvements in enzymes toward their biotechnological applications. *Front. Microbiol.* 7:965 10.3389/fmicb.2016.00965 PMC4909775 27379087

[B7] Bent A. F. Mackey D. (2007). Elicitors, effectors, and r genes: the new paradigm and a lifetime supply of questions. *Annu. Rev. Phytopathol.* 45 399–436. 17506648 10.1146/annurev.phyto.45.062806.094427

[B8] Bergelson J. Kreitman M. Stahl E. A. Tian D. (2001). Evolutionary dynamics of plant R-genes. *Science* 292 2281–2285. 10.1126/science.1061337 11423651

[B9] Bonman J. M. Vergel de Dios T. I. Khim M. M. (1986). Physiologic specialization of *Pyricularia oryzae* in the Philippines. *Plant Dis.* 70 767–769. 10.1094/PD-70-767

[B10] Collier S. M. Moffett P. (2009). NB-LRRs work a “bait and switch” on pathogens. *Trends Plant Sci.* 14 521–529. 10.1016/j.tplants.2009.08.001 19720556

[B11] Dai Y. Jia Y. Correll J. Wang X. Wang Y. (2010). Diversification and evolution of the avirulence gene AVR-Pita1 in fields isolate of *Magnaporthe oryzae*. *Fungal Genet. Biol.* 47 973–980. 10.1016/j.fgb.2010.08.003 20719251

[B12] Ding J. Zhang W. Jing Z. Chen J. Q. Tian D. (2007). Unique pattern of *R*-gene variation within populations in *Arabidopsis*. *Mol. Genet. Genomics* 277 619–629. 10.1007/s00438-007-0213-5 17277944

[B13] Ewing B. Green P. (1998). Base calling sequencer traces using Phred II. Error probabilities. *Genome Res.* 8 175–185. 10.1101/gr.8.3.175 9521922

[B14] Geng X. S. Yang M. Z. Huang X. Q. Cheng Z. Q. Fu J. Sun T. (2008). Cloning and analyzing of rice blast resistance gene Pi-ta^+^ allele from Jinghong erect type of common wild rice (*Oryza rufipogon* Griff) in Yunnan. *Yi Chuan* 30 109–114. 10.3724/SP.J.1005.2008.00109 18244911

[B15] Gupta P. K. Rustogi S. (2004). Molecular markers from the transcribed/expressed region of the genome in higher plants. *Funct. Integr. Genomics* 4 139–162. 10.1007/s10142-004-0107-0 15095058

[B16] Hammond-Kosack K. E. Jones J. D. G. (1997). Plant disease resistance genes. *Plant Mol. Biol.* 48 575–607. 10.1146/annurev.arplant.48.1.575 15012275

[B17] Han S. S. Ryu J. D. Shim H. S. Lee S. W. Hong Y. K. Cha K. H. (2001). Breakdown of resistant cultivars by new race KI-1117a and race distribution of rice blast fungus during 1999-2000 in Korea. *Res. Plant Dis.* 7 86–92.

[B18] Huang E. Hwang S. Chiang Y. Lin T. (2008). Molecular evolution of the Pi-ta gene resistant to rice blast in wild rice (*Oryza rufipogon*). *Genetics* 179 1527–1538. 10.1534/genetics.108.089805 18622033 PMC2475752

[B19] Hudson R. Kreitman R. M. Aguadé M. (1987). A test of neutral molecular evolution based on nucleotide data. *Genetics* 116 153–159. 3110004 10.1093/genetics/116.1.153 PMC1203113

[B20] Imam J. Alam S. Mandal N. P. Maiti D. Variar M. Shukla P. (2014a). Molecular diversity and mating type distribution of the rice blast pathogen *Magnaporthe oryzae* in North-east and Eastern India. *Ind. J. Microbiol.* 55:108 10.1007/s12088-014-0504-6

[B21] Imam J. Alam S. Mandal N. P. Shukla P. Sharma T. R. Variar M. (2015a). Molecular identification and virulence analysis of AVR genes in rice blast pathogen, *Magnaporthe oryzae* from Eastern India. *Euphytica* 206 21–31.

[B22] Imam J. Alam S. Mandal N. P. Variar M. Shukla P. (2014b). Molecular screening for identification of blast resistance genes in North East and Eastern Indian rice germplasm (*Oryza sativa* L.) with PCR based markers. *Euphytica* 196 199–211. 10.1007/s10681-013-1024-x

[B23] Imam J. Alam S. Variar M. Shukla P. (2013a). Identification of rice blast resistance gene Pi9 from Indian rice landraces with STS marker and its verification by virulence analysis. *Proc. Natl. Acad. Sci. India Sect. B Biol. Sci.* 83 499–504. 10.1007/s40011-013-0186-6

[B24] Imam J. Mahto D. Mandal N. P. Maiti D. Shukla P. Variar M. (2014c). Molecular analysis of indian rice germplasm accessions with resistance to blast pathogen. *J. Crop Improv.* 28 1–11.

[B25] Imam J. Mandal N. P. Variar M. Shukla P. (2015b). “Advances in molecular mechanism toward understanding plant-microbe interaction: A study of *M. oryzae* Vs rice,” in *Frontier Discoveries and Innovations in Interdisciplinary Microbiology* ed. Shukla P. (New Delhi: Springer) 79–96.

[B26] Imam J. Nitin M. Toppo N. N. Mandal N. P. Kumar Y. Variar M. (2014d). “A comprehensive overview on application of bioinformatics and computational statistics in rice genomics towards an amalgamated approach for improving acquaintance base,” in *Agricultural Bioinformatics* Chap. 5 eds Kishor P. B. K. Bandyopadhyay R. Suravajhala P. (Berlin: Springer).

[B27] Imam J. Singh P. K. Shukla P. (2016). Plant microbe interactions in post genomic era: perspectives and applications. *Front. Microbiol.* 7:1488 10.3389/fmicb.2016.01488 PMC5035750 27725809

[B28] Imam J. Variar M. Shukla P. (2013b). “Role of enzymes and proteins in plant-microbe interaction: a study of *M. oryzae* versus rice,” in *Advances in Enzyme Biotechnology* Vol. 10 eds Shukla P. Pletschke B. I. (Berlin: Springer) 137–145.

[B29] Jia Y. Martin R. (2008). Identification of a new locus Ptr(t) required for rice blast resistance gene Pi-ta-mediated resistance. *Mol. Plant Microbe Interact.* 21 396–403. 10.1094/MPMI-21-4-0396 18321185

[B30] Khush G. S. Jena K. K. (2009). “Current status and future prospects for research on blast resistance in rice (Oryza sativa L.),” in *Advances in Genetics, Genomics and Control of Rice Blast Disease* eds Wang G.-L. Valent B. (Dordrecht: Springer Science+Business Media B.V.) 1–10.

[B31] Kiyosawa S. Mackill D. J. Bonman J. M. Tanaka Y. Ling Z. Z. (1986). An attempt of classification of world’s rice varieties based on reaction pattern to blast fungus strains. *Bull. Natl. Inst. Agrobiol. Resour.* 2 13–39.

[B32] Kumar G. R. Sakthivel K. Sundaram R. M. Neeraja C. N. Balachandran S. M. Rani N. S. (2010). Allele mining in crops: prospects and potentials. *Biotechnol. Adv.* 18 451–461. 10.1016/j.biotechadv.2010.02.007 20188810

[B33] Kumar V. Baweja M. Singh P. K. Shukla P. (2016). Recent developments in systems biology and metabolic engineering of plant microbe interactions. *Front. Plant Sci.* 7:1421 10.3389/fpls.2016.01421 PMC5035732 27725824

[B34] Kwon J. O. Lee S. G. (2002). Real-time micro-weather factors of growing field to the epidemics of rice blast. *Res Plant Dis.* 8 199–206. 10.5423/RPD.2002.8.4.199

[B35] Lee S. Costanzo S. Jia Y. Olsen K. M. Caicedo A. L. (2009). Evolutionary dynamics of the genomic region around the blast resistance gene Pi-ta in AA genome *Oryza* species. *Genetics* 183 1315–1325. 10.1534/genetics.109.108266 19822730 PMC2787423

[B36] Lee S. Jia Y. Jia M. Gealy D. R. Olsen K. M. (2011). Molecular evolution of the rice blast resistance gene Pita in invasive weedy rice in the USA. *PLoS ONE* 6:e26260 10.1371/journal.pone.0026260 PMC3197024 22043312

[B37] Li Y. B. Wu C. J. Jiang G. H. Wang L. Q. He Y. Q. (2007). Dynamic analyses of rice blast resistance for the assessment of genetic and environmental effects. *Plant Breed.* 126 541–547. 10.1111/j.1439-0523.2007.01409.x

[B38] Liu J. Hu Y. Ning Y. Jiang N. Wu J. Xiao Y. (2011). Genetic variation and evolution of the Pi9 blast resistance locus in the AA genome *Oryza* species. *J. Plant Biol.* 54 294–302. 10.1007/s12374-011-9166-7

[B39] Liu J. Wang X. Mitchell T. Hu Y. Liu X. Dai L. (2010). Recent progress and understanding of the molecular mechanisms of the rice-*Magnaporthe oryzae* interaction. *Mol. Plant Pathol.* 11 419–427. 10.1111/j.1364-3703.2009.00607.x 20447289 PMC6640493

[B40] Liu X. Lin F. Wang L. Pan Q. (2007). The in silico map-based cloning of Pi36, a rice coiled-coil nucleotide-binding site leucine-rich repeat gene that confers race-specific resistance to the blast fungus. *Genetics* 176 2541–2549. 10.1534/genetics.107.075465 17507669 PMC1950653

[B41] Liu X. Q. Wang L. Chen S. Lin F. Pan Q. H. (2005). Genetic and physical mapping of Pi36(t), a novel rice blast resistance gene located on rice chromosome 8. *Mol. Genet. Genomics* 274 394–401. 10.1007/s00438-005-0032-5 16151856

[B42] Liu Y. Liu B. Bordeos A. Leung H. Zhu X. Wang G. (2013). Fine-mapping and molecular marker development for Pi56(t), a NBS-LRR gene conferring broad-spectrum resistance to *Magnaporthe oryzae* in rice. *Theor. Appl. Genet.* 126 985–998. 23400829 10.1007/s00122-012-2031-3

[B43] MacKill D. J. Bonman J. M. (1992). Inheritance of blast resistance in near-isogenic lines of rice. *Phytopathology* 82 746–749. 10.1094/Phyto-82-746

[B44] McDonald J. H. Kreitman M. (1991). Adaptive evolution at the Adh locus in *Drosophila*. *Nature* 351 652–654. 10.1038/351652a0 1904993

[B45] Qu S. Liu G. Zhou B. Bellizz I. M. Zeng L. Dai L. (2006). The broad-spectrum blast resistance gene Pi9 encodes a nucleotide-binding site_leucine-rich repeat protein and is a member of a multigene family in rice. *Genetics* 172 1901–1914. 10.1534/genetics.105.044891 16387888 PMC1456263

[B46] Rakshit S. Rakshit A. Matsumura H. Takahashi Y. Hasegawa Y. Ito A. (2007). Large scale DNA polymorphism study of *Oryza sativa* and *Oryza rufipogon* reveals the origin and divergence of Asian rice. *Theor. Appl. Genet.* 114 731–743. 10.1007/s00122-006-0473-1 17219210

[B47] Rozas J. Sańchez-DelBarrio J. C. Messeguer X. Rozas R. (2003). DnaSP, DNA polymorphism analyses by the coalescent and other methods. *Bioinformatics* 19 2496–2507. 10.1093/bioinformatics/btg359 14668244

[B48] Saitou N. Nei M. (1987). The neighbour-joining method: a new method for reconstructing phylogenetic trees. *Mol. Biol. Evol.* 4 406–425. 3447015 10.1093/oxfordjournals.molbev.a040454

[B49] Shang J. Tao Y. Chen X. Zou Y. Lei C. Wang J. (2009). Identification of a new rice blast Resistance gene, Pid3, by genome wide comparison of paired nucleotide-binding site leucine-rich repeat genes and their pseudogene alleles between the two sequenced rice genomes. *Genetics* 182 1303–1311. 19506306 10.1534/genetics.109.102871 PMC2728867

[B50] Sharma T. R. Madhav M. S. Singh B. K. Shanker P. Jana T. K. Dalal V. (2005). High resolution mapping, cloning and molecular characterization of the Pi-kh gene of rice, which confers resistance to M. *grisea*. *Mol. Genet. Genomics* 274 569–578. 10.1007/s00438-005-0035-2 16228246

[B51] Shen J. H. Araki H. Chen L. Chen J. Q. Tian D. (2006). Unique evolutionary mechanism in R-genes under the presence/absence polymorphism in *Arabidopsis thaliana*. *Genetics* 172 1243–1250. 10.1534/genetics.105.047290 16452149 PMC1456222

[B52] Tajima F. (1989). Statistical method for testing the neutral mutation hypothesis by DNA polymorphism. *Genetics* 123 585–595. 2513255 10.1093/genetics/123.3.585 PMC1203831

[B53] Takken F. L. Tameling W. I. (2009). To nibble at plant resistance proteins. *Science* 324 744–746. 10.1126/science.1171666 19423813

[B54] Tamura K. Dudley J. Nei M. Kumar S. (2007). MEGA4: molecular evolutionary genetics analysis (MEGA) software version 4.0. *Mol. Biol. Evol.* 24 1596–1599. 10.1093/molbev/msm092 17488738

[B55] Teng P. S. Klein-Debbinck H. W. Pinnschmidt H. (1991). “An analysis of the blast pathosystem to guide modelling and foresting,” in *Proceedings of the Selected papers from the International Rice Research Conference: Rice Blast Modeling and Forecasting* (Manila: IRRI) 2–5.

[B56] Thakur S. Singh P. K. Das A. Rathour R. Variar M. Prashanthi S. K. (2015). Extensive sequence variation in rice blast resistance gene Pi54 makes it broad spectrum in nature. *Front. Plant Sci.* 6:345 10.3389/fpls.2015.00345 PMC4440361 26052332

[B57] Thakur S. Singh P. K. Rathour R. Variar M. Prashanthi S. K. Singh A. K. (2013). Positive selection pressure on rice blast resistance allele Piz-t makes it divergent in Indian landraces. *J. Plant Interact.* 8 34–44. 10.1080/17429145.2012.721523

[B58] Thompson J. D. Higgins D. G. Gibson T. J. (1994). CLUSTALW: improving the sensitivity of progressive sequence alignment through sequence weighting, position-specific gaps penalties and weight matrix choice. *Nucleic Acids Res.* 22 4673–4680. 10.1093/nar/22.22.4673 7984417 PMC308517

[B59] Till B. J. Reynolds S. H. Green E. A. Codomo C. A. Enns L. C. Johnson J. E. (2003). Large-scale discovery of induced point mutations with high-throughput TILLING. *Genome Res.* 13 524–530. 10.1101/gr.977903 12618384 PMC430291

[B60] Valent B. (1990). Rice blast as a model system for plant pathology. *Phytopathology* 80 33–36. 10.1094/Phyto-80-33

[B61] Valent B. Chumley F. G. (1994). “A virulence genes and mechanism of genetic instability in the rice blast fungus,” in *Rice Blast Disease* eds Zeigler R. S. Leong S. A. Teng P. S. (Wallingford, CT: CAB International) 111–134.

[B62] Variar M. Vera Cruz C. M. Carrillo M. G. Bhatt J. C. Sangar R. B. S. (2009). “Rice blast in India and strategies to develop durably resistant cultivars,” in *Advances in Genetics, Genomics and Control of Rice Blast Disaese* eds Wang X. Valent B. (Berlin: Springer) 359–374.

[B63] Xia J. Q. Correll J. C. Lee F. N. Marchetti M. A. Rhoads D. D. (1993). DNA fingerprinting to examine microgeographic variation in the *Magnaporthe grisea* (*Pyricularia grisea*) population in two rice fields in Arkansas. *Phytopathology* 83 1029–1035. 10.1094/Phyto-83-1029

[B64] Yang S. Feng Z. Zhang X. Jiang K. Jin X. Hang Y. (2006). Genome-wide investigation on the genetic variations of rice disease resistance genes. *Plant Mol. Biol.* 62 181–193. 10.1007/s11103-006-9012-3 16915523

[B65] Yang S. Gu T. Pan C. Feng Z. Ding J. Hang Y. (2008). Genetic variation of NBS-LRR class resistance genes in rice lines. *Theor. Appl. Genet.* 16 165–177. 10.1007/s00122-007-0656-4 17932646

[B66] Young B. J. D. Innes R. W. (2006). Plant NBS-LRR proteins in pathogen sensing and host defense. *Nat. Immunol.* 7 1243–1249. 10.1038/ni1410 17110940 PMC1973153

[B67] Zhou B. Dolan M. Sakai H. Wang G. L. (2007). The genomic dynamics and evolutionary mechanism of the Pi2/9 locus in rice. *Mol. Plant Microbe Interact.* 20 63–71. 10.1094/MPMI-20-0063 17249423

[B68] Zhou B. Qu S. Liu G. Dolan M. Sakai H. Lu G. (2006). The eight amino-acid differences within three leucine-rich repeats between Pi2 and Piz-t resistance proteins determine the resistance specificity to *Magnaporthe grisea*. *Mol. Plant Microbe Interact.* 19 1216–1228. 10.1094/MPMI-19-1216 17073304

